# Use of Intraoperative Low‐Dose Glucocorticoids in Patients With Abdominal Sepsis Undergoing Surgery

**DOI:** 10.1002/hsr2.71014

**Published:** 2025-07-09

**Authors:** Kinza Irshad, Ashfaq Ahmad, Javed Iqbal

**Affiliations:** ^1^ Ziauddin University Karachi Pakistan; ^2^ Gomal Medical College D.I.Khan Dera Ismail Khan Pakistan; ^3^ Nursing Department Hamad Medical Corporation Doha Qatar

## Author Contributions

**Kinza Irshad:** conceptualization, investigation, methodology, data curation, writing – review and editing. **Ashfaq Ahmad:** Investigation, conceptualization, methodology, validation, data curation, supervision, writing – review and editing. **Javed Iqbal:** conceptualization, investigation, funding acquisition, supervision, resources, and validation.

To the Editor,

I have read with interest the article, “*Intraoperative Low‐Dose Glucocorticoids in Surgical Patients With Abdominal Sepsis: A Multicenter Retrospective Cohort Study*” by Tianzhu Tao et al. [1], and I want to commend and congratulate the authors for their impressive and committed work in establishing a significant medical correlation between administering glucocorticoids during surgery to patients with diagnosed abdominal sepsis and how it affects their clinical results. Sepsis is a lethal disease state that results in organ failure, and it is caused by impaired or heightened host responses to an infection, resulting in critical illness that subjects the patients to an intensive care unit (ICU) to support their vital organs [2]. Glucocorticoids are potent medications used as the primary drugs of choice in inflammatory diseases as they have effective anti‐inflammatory properties [3]. However, I would like to add that it is worth noting that one major emerging issue associated with prolonged high circulating glucocorticoid (GC) concentrations is the development of GC resistance [3]. Some sepsis‐related clinical studies have suggested a peripheral decrease in expression of the active glucocorticoid receptor (GRα) gene in blood, which could be seen as the marker of “generalized resistance” to glucocorticoids during sepsis [2]. These elements might affect GC signalling and lessen the effectiveness of GCs, and make individuals respond to it even less, raising the likelihood of septic shock and postoperative mortality, even with the use of GCs during surgery.

Pathogen‐associated molecular patterns (PAMPs) and damage‐associated molecular patterns (DAMPs) are stimulated by host immune responses during sepsis [2]. All participants involved in this study underwent extensive surgery that could have triggered hyperinflammation similar to sepsis via induction of DAMPs [2], that also trigger the host immune responses, therefore making the sepsis‐induced critical illness even worse [2]. These factors can worsen the predicted disease course, creating variance in individual clinical outcomes and increasing the rate of mortality.

Although the research highlighted the utility of the SOFA score for diagnosis of sepsis, it failed to address any laboratory assessments conducted for sepsis biomarkers including acute‐phase proteins, cytokines, chemokines, DAMPs, endothelial cell markers, leukocyte surface markers, noncoding RNAs, miRNA, and soluble receptors, as well as metabolites and alterations in gene expression (transcriptomics) [4]. Research shows that these biomarkers could assist in categorizing patients undergoing sepsis into various biological phenotypes, such as hyperinflammatory versus immunosuppressive [4], further guiding the prognosis and patient‐specific treatments. These biological biomarkers can also serve as a tool to assess a patient's gut as well as blood‐brain barrier permeability, chances of being readmitted into the hospital, and longer‐term clinical results [4].

Personalized cell‐type and individual‐specific treatment strategies should have been preferred as individual differences in genetics and epigenetics can impact GC metabolism, responsiveness as well as adverse effects. Genetic variations in the form of single‐nucleotide polymorphisms (SNPs) are statistically linked to complex diseases and modulate the individual's response to drugs [5]. One study in a mouse model showed decreased death risk after an induction of faulty hepatic GRα signaling [2]. A different research depicted that partly declining the expression of GRα, especially in the liver, via small hairpin RNA, can cause widespread hyperinflammation throughout the body, resulting in poor prognosis of septic cases [2].

Finally, though synthetic glucocorticoids when taken for a short period of time vastly help in reducing inflammation, chronic exposure can cause immunosuppression [3], thus they might diminish the body′s inherent immune defenses, increasing the risk of secondary infections and, consequently, mortality rate and hospital stay. Higher doses (typically > 5 mg/day) of synthetic GC are linked to various adverse side effects such as insulin resistance and, therefore, an increase in blood sugar levels, high blood pressure, weight gain, stunted growth, osteoporosis, and anxiety [3], predisposing patients to additional stressors. Therefore, the negative impacts of glucocorticoids should not be overlooked, particularly when given to patients in critical conditions and when assessing long‐term clinical results.

## Data Availability

The data that support the findings of this study are available on request from the corresponding author. The data are not publicly available due to privacy or ethical restrictions.

## References

[1] T. Tao , Y. Shi , X. Ye , W. Mi , and J. Lou , “Intraoperative Low‐Dose Glucocorticoids In Surgical Patients With Abdominal Sepsis: A Multicenter Retrospective Cohort Study,” Health Science Reports 8 (2025): e70360, 10.1002/hsr2.70360. 39980824 PMC11839392

[2] G. Van den Berghe , A. Téblick , L. Langouche , and J. Gunst , “The Hypothalamus‐Pituitary‐Adrenal Axis In Sepsis‐ and Hyperinflammation‐Induced Critical Illness: Gaps in Current Knowledge and Future Translational Research Directions,” EBioMedicine 84 (2022): 104284, 10.1016/j.ebiom.2022.104284. 36162206 PMC9519475

[3] G. J. Martinez , M. Appleton , Z. A. Kipp , A. S. Loria , B. Min , and T. D. Hinds Jr. , “Glucocorticoids, Their Uses, Sexual Dimorphisms, and Diseases: New Concepts, Mechanisms, and Discoveries Genesee Glucocorticoids, Their Uses, Sexual Dimorphisms, and Diseases: New Concepts, Mechanisms, and Discoveries,” Physiological Reviews 104, no. 1 (2024): 473–532. 37732829 10.1152/physrev.00021.2023 PMC11281820

[4] T. Barichello , J. S. Generoso , M. Singer , and F. Dal‐Pizzol , “Biomarkers for Sepsis: More Than Just Fever and Leukocytosis—A Narrative Review,” Critical Care 26 (2022): 14, 10.1186/s13054-021-03862-5. 34991675 PMC8740483

[5] W. Hu , C. Jiang , M. Kim , et al., “Individual‐Specific Functional Epigenomics Reveals Genetic Determinants of Adverse Metabolic Effects of Glucocorticoids,” Cell Metabolism 33, no. 8 (August 2021): 1592–1609.e7, 10.1016/j.cmet.2021.06.004. 34233159 PMC8340270

